# Low 25(OH)-vitamin D concentrations are associated with emotional and behavioral problems in German children and adolescents

**DOI:** 10.1371/journal.pone.0183091

**Published:** 2017-08-23

**Authors:** Christiane Husmann, Mirjam Frank, Börge Schmidt, Karl-Heinz Jöckel, Jochen Antel, Volker Reissner, Lars Libuda, Johannes Hebebrand, Manuel Föcker

**Affiliations:** 1 Department of Child and Adolescent Psychiatry, University Hospital Essen, University Duisburg-Essen, Essen, Germany; 2 Institute for Medical Informatics, Biometry und Epidemiology, University Hospital Essen, University Duisburg-Essen, Essen, Germany; TNO, NETHERLANDS

## Abstract

**Background:**

Evidence has accumulated for the association between low vitamin D serum concentrations and mental health disorders in both children and adults. We performed a cross-sectional analysis in a population-based sample of children and adolescents to detect associations between 25(OH)-vitamin D serum [25(OH)D] concentrations and scores of the five Strengths and Difficulties Questionnaire (SDQ) subscales and the total difficulties score in different age groups (age ≥3-<12 years and ≥12-<18 years).

**Methods:**

9068 participants of the population-based, nation-wide German Health Interview and Examination Survey for Children and Adolescents (KIGGS) with information on mental health status assessed by the SDQ and 25(OH)D levels were included in the analysis. For statistical analysis we used linear regression models stratified by gender based on different adjustment sets. For the younger subsample the analysis was additionally adjusted for the frequency of playing outside. We compared the associations based on parent- and self-ratings of the SDQ for children and adolescents aged ≥12-<18 years.

**Results:**

We found inverse associations between 25(OH)D concentrations and the subscales emotional problems, peer relationship problems and the total difficulties score in both genders after adjustment for potential confounders. The strongest associations were observed in the older subsample for parent-ratings in boys and self-ratings in girls. In the younger subsample the associations were less strong and no longer evident after adjustment for potential confounders such as migration background, socioeconomic status and frequency of playing outside.

**Conclusion:**

Based on the large-scale cross-sectional study in a German population-based sample of children and adolescents we detected inverse associations between 25(OH)D concentrations and both parent- and self-rated SDQ scores of the total difficulties scale and different subscales with the strongest association in the subsample aged ≥12-<18 years for both genders. Migration background and socioeconomic status were detected as relevant confounders. Further studies–particularly in countries with comparatively low mean 25(OH)D concentrations–in childhood and adolescence are warranted. Longitudinal studies are also necessary to infer direction of effects. Finally, RCTs in children and adolescents are required to determine whether Vitamin D is beneficial for mental health.

## Introduction

1,25-dihydroxyvitamin D (calcitriol), the metabolic product of vitamin D [1], is a pleiotropic secosteroid hormone targeting hundreds of genes [2]. The vitamin D status is usually [3] assessed via the total 25(OH)-vitamin D [25(OH)D] serum concentration, which is mainly a product of 25(OH)D_3_ from ultraviolet B radiation (UVB) induced synthesis in the skin and to a lesser extent from dietary intake of 25(OH)D_2_ [4].

1,25-dihydroxyvitamin D_3_ receptor (VDR) and 25-hydroxyvitamin D_3_ 1-alpha-hydroxylase are localized throughout the brain [5], suggesting a role for vitamin D in brain functions. In adults as well as in children and adolescents, associations between 25(OH)D deficiency and mental disorders such as depression, autism and ADHD have been reported [6–15]. Meta-analyses of randomized controlled trials (RCTs) of adult psychiatric patients with both major depression and vitamin D deficiency found poor evidence for a beneficial effect of vitamin D [16–19]. However, upon meta-analysis of only those seven studies [20–26] without “biological flaws” according to Spedding [17], a convincing effect-size (standardized mean difference = 0.78; CI 0.24–1.27) suggested an antidepressant effect of vitamin D supplementation. To our knowledge, only one RCT with methodological limitations was performed in children and adolescents; no significant effect after 6 months of vitamin D supplementation on Autism Spectrum Disorder (ASD) outcome scores was found [27]. However, vitamin D supplementation trials (not a RCT) found a positive effect of vitamin D supplementation on ASD and depression in children [28–30].

The relationship between 25(OH)D_2_, 25(OH)D_3_, and total 25(OH)D serum levels and mental health problems in a population-based sample of children was for the first time investigated in the Avon Longitudinal Study of Parents & Children (ALSPAC), a birth cohort study from South West England [31–34]. 25(OH)D_3_ accounted for 94.4% (median) of the total 25(OH)D in the ALSPAC cohort [31]. In this cohort 29% of the children (mean age of 9.9 years) [31] had 25(OH)D concentrations below the cut-off of 50 nmol/l recommended by the Institute of Medicine (IoM) [35].

In contrast, in the German National Health Interview and Examination Survey for Children and Adolescents (KiGGS) 69% of the children (aged seven to 13 years) exhibited a 25(OH)D concentration below the IoM cut-off [36]. This difference might be partly explained by the fact that within the time frame of the ALSPAC study, vitamin D fortification of e.g. margarine and cereals was mandatory in the United Kingdom [31]. The higher prevalence of vitamin D deficiency could make potential associations with mental health problems even more relevant in children and adolescents in Germany than in the ALSPAC cohort. Here, the association of 25(OH)D_2_/ 25(OH)D_3_ serum levels determined at a mean age of 9.8 years with self-rated depressive symptoms assessed by the Mood and Feelings Questionnaire at mean ages of 10.6 (n = 2.759) and 13.8 years (n = 2.752) was analyzed adjusted for several potential confounders (i.e. ethnicity, age, gender, head of household occupational social class, maternal and paternal education, time spent outdoors during summer (age 8.5 years), UVB protection score, WISC IQ score at 8.5 years, BMI, family history of depression or schizophrenia, puberty stage, serum concentrations of phosphate, albumin-adjusted calcium, and parathyroid hormone) [32]. An association between higher concentrations of season-adjusted 25(OH)D_3_ and lower levels of depressive symptoms was only detected at the mean age of 13.8 years, thus, suggesting an effect that showed up only after a longer follow-up period [32]. In another prospective analysis of the ALSPAC cohort, the investigators detected an inverse association between adjusted total serum 25(OH)D_3_ concentrations and definite psychotic experiences [34].

However, another analysis of the ALSPAC cohort did not provide conclusive evidence of a robust association between emotional/behavioral problems and vitamin D levels in children and adolescents and contrasted somewhat with the above-mentioned associations. In this analysis, parent-ratings of the Strengths and Difficulties Questionnaire (SDQ) obtained at mean ages of 7.6, 9.6, and 11.7 years were related to the 25(OH)D_2_ and 25(OH)D_3_ serum concentrations at the mean age of 9.9 years [33] again adjusted for the same potential confounders [32]. This analysis showed that serum 25(OH)D_3_ and 25 (OH)D_2_ concentrations at a mean age of 9.6 years were not significantly associated with incident behavioral problems as assessed with the SDQ total difficulties score at a mean age of 11.7 years; however a trend in the expected direction was apparent (odds ratios of approximately 0.9 in all three models; the 95^th^ confidence intervals ranged from 0.75 to 1.02). Higher 25(OH)D_3_ and lower 25(OH)D_2_ concentrations were weakly associated with a lower risk of "prosocial problems", but not with the subscales emotional symptoms, conduct problems, hyperactivity, and peer relationship problems.

In a cross sectional analysis of the 11–17 year old KIGGS subsample [37] significant inverse associations of the SDQ total difficulties score, emotional problems and peer relationship problems with serum 25(OH)D levels in self- and parent ratings were found according to an unadjusted bivariate generalized linear model; the effect size estimates were comparably small. In additional multifactorial models based on the SDQ total difficulties score as dependent variable, 25(OH)D serum level as independent variables and several covariables (Model 1: age, sex, BMI, mean systolic blood pressure, migration background, and SES) revealed that the negative association remained stable irrespective of the use of self- or parent ratings. However, if screen time was additionally included in the analysis, the effect size estimate for the self-rated total difficulties score no longer remained significant.

Considering the partly conflicting results obtained by Tolppanen et al. [31–34] and Schäfer et al. [37], the aim of the present study was to investigate cross-sectional associations between total 25(OH)D concentrations and mental health problems, determined with the SDQ in the nation-wide KiGGS study population across the completely available age range from 3 to 18 years. We analyzed the influence of potential confounders/ mediators on this association. Furthermore, we compared associations based on different age groups (age ≥3-<12 years and ≥12-<18 years) and based on self- and parent-reported behavioral symptoms for the age group ≥12-<18 years in light of the well-known discrepancies in ratings of problem behavior between the two informants children/adolescents and parents, respectively [38].

## Materials and methods

### Study population

The current study made use of cross-sectional data collected during the baseline examination (May 2003—May 2006) of the population-based and nation-wide KiGGS study, which was carried out in 167 German communities. Overall, n = 17.641 children aged >0 to ≤18 years, randomly sampled from local population registries, were enrolled. The survey comprised self-administered questionnaires, personal interviews and a medical examination to assess somatic and mental health issues (for details see Hölling et al. 2008 [39]). Informed consent was obtained from both, children and parents.

Children younger than three years (n = 2,805) were excluded from our study sample, because data on mental health had not been collected for this age group. Additionally, we excluded children with missing measures of serum 25(OH)D concentrations and/or missing information on all SDQ subscales (n = 5,767). Thus, the present analysis is based on a sample of 9,068 participants aged 3–17.9 years. The excluded participants did not substantially differ from the selected study population in terms of gender, and risk factors under investigation. For all sub-analyses pertaining to the item “frequency of playing outside” (see below), only children aged 3–11.9 years were included (n = 4632), as the respective question was asked in this age group only.

### Data collection

Data was based on age-specific standardized self-administered questionnaires for parents and also for children aged 11 years and older and physical examinations [39].

Mental health problems were assessed with the SDQ to obtain scores (range 0 to 10) for the five subscales (emotional problems, hyperactivity/inattention, conduct problems, peer relationship problems and prosocial behavior) inserted as continuous variables into regression analyses. The total difficulties score was calculated as a sum of the scores of four SDQ subscales (range 0 to 40), excluding the subscale prosocial behavior [40]. Information on mental health was based on parental SDQ only for children aged ≥3-<12 years and ≥12-<18 years, while the ≥12- <18 year-olds in addition self-rated their emotional and behavioral symptoms, thus allowing two regression analyses for this older age group.

Venous blood samples from non-fasting subjects were obtained and immediately processed and separated. Total 25(OH)D serum concentrations were assessed with Luminescence Immunoassay [(LIA, DiaSorin Deutschland GmbH, Dietzenbach)] [41, 42].

Socioeconomic status (SES) classification was based on a combination of different indicators (household income, parental occupation and educational attainment) and rated using a scoring system (1 to 7 points per indicator). A continuous variable was used for regression analyses, whereas a categorical variable was created for descriptive analyses. Three categories were assigned: low (3–8 points), middle (9–14) or high (15–21) parental SES [43].

Study participants were referred to as having a migration background if they themselves were immigrants or had at least one parent, who had immigrated or was of non-German nationality [44].

Trained staff carried out the anthropometric measurements. Children's height was measured without wearing shoes, with an accuracy of 0.1 cm. Body weight was measured with an accuracy of 0.1 kg, wearing underwear. These measures were used to calculate BMI in kg/m^2^. The weight status was classified using age-and gender-specific BMI percentiles (P) [45] as severe underweight (P<3), underweight (P3 - <P10), normal weight (P10- P90), overweight (>P90) and obese (>P97).

Information on sexual maturation status was obtained in children older than eight years by self-assessment of pubic hair status according to standardized drawings. The maturation status was classified into six Tanner stages, ranging from Tanner stage 1 describing infantile children to Tanner stage 6 describing post pubertal maturation [46] and induced into calculations as dummy variables with Tanner stage 1–3 as reference. Children under eight years were assigned to Tanner stage 1.

Parents of children aged ≥3-<12 years were asked about the frequency of their child playing outside (answer categories were: “almost daily”, “3–5 times per week”, “1–2 times per week”, “seldom” or “never”[47]).

### Statistical analyses

Descriptive statistics were computed stratified by gender. Continuous variables are reported as means and standard deviations (SD), and categorical variables are given in frequency and percent.

For the analysis of children and adolescents aged ≥3-<18 the association of 25(OH)D concentrations (independent variable) with mental health problems (dependent variable) was analyzed using linear regression models stratified by gender with different adjustment sets as follows: age-adjusted (Model a), age- and SES-adjusted (Model b), age- and migration status-adjusted (Model c), fully adjusted (i.e. age-, SES-, migration status-, BMI-, Tanner stages-adjusted) (Model d).

The same adjustment sets were fitted for a subsample of children aged 3–11.9. These adjustment sets were extended by the additional variable “frequency of playing outside”, which was included as a dichotomous variable (almost daily vs. all others). To compare the effects in adolescents with those in children regression analysis was also performed in the older subsample (12–17.9 years).

All analyses were performed using SPSS software (Version 22, SPSS GmbH Software).

## Results

### Description of study population

Characteristics of the total study population are given in Table 1. The parent-rated SDQ mean total difficulties scores of the total sample were within the normal range (<14) as expected for a large population based sample. The mean 25(OH)D concentrations in boys and girls were approximately 1.5–3.5 nmol/l, which is below the threshold concentration of 50 nmol/l (= 20 ng/mL) recommended by the IoM.

**Table 1:** Characteristics of study population aged 3-17years.

		Boys	Girls
**N**		4623	4445
**Age (Years)**^a^			
3		232 (5.0%)	232 (5.2%)
4–5		529 (11.4%)	519 (11.7%)
6–7		626 (13.5%)	588 (13.2%)
8–9		677 (14.6%)	643 (14.5%)
10–11		675 (14.6%)	644 (14.4%)
12–13		682 (14.8%)	633 (14.2%)
14–15		665 (14.4%)	617 (13.8%)
16–17		537 (11.6%)	569 (12.7%)
**25(OH)Vitamin D [nmol/l]** ^b^		46.8 ± 25.0	46.5 ± 25.5
**Strengths and Difficulties Questionnaire, parent-ratings**^b^			
Emotional Problems		1.7 ± 1.8	1.8 ± 1.8
Conduct Problems		2.0 ± 1.6	1.8 ± 1.5
Hyperactivity		3.5 ± 2.3	2.7 ± 2.1
Peer Relationship Problems		1.5 ± 1.7	1.3 ± 1.5
Prosocial Behaviour		7.6 ± 1.8	8.1 ± 1.6
Total Difficulties Score		8.7 ± 5.3	7.6 ± 4.9
**Socioeconomic Status**^a^			
Low		1270 (27.7%)	1192 (27.0%)
Middle		2167 (47.3%)	2084 (47.3%)
High		1143 (25.0%)	1134 (25.7%)
**Migration Background**^a^		378 (13.8%)	578 (13.0%)
**Body Mass Index (Percentiles)**^a^			
Severely Underweight (<P3)		96 (2.1%)	77 (1.7%)
Underweight (P3- <P10)		249 (5.4%)	227 (5.1%)
Normal (Healthy Weight)		3568 (77.5%)	3476 (78.5%)
Overweight (>P90 –P97)		406 (8.8%)	384 (8.8%)
Obese (>P97)		285 (6.2%)	263 (5.9%)
**Growth of Pubic Hair (Tanner Stages)**^a^	1	2410 (52.7%)	2285 (51.8%)
	2	492 (10.6%)	231 (5.2%)
	3	268 (5.8%)	189 (4.3%)
	4	346 (7.5%)	561 (12.7%)
	5	721 (15.6%)	885 (20.2%)
	6	336 (7.3%)	256 (5.8%)

^a^ n(%)

^b^ Mean (Standard deviation)

### Linear regression analysis of total 25(OH)-vitamin D concentration and SDQ total difficulties and subscale scores

**Fig 1:**
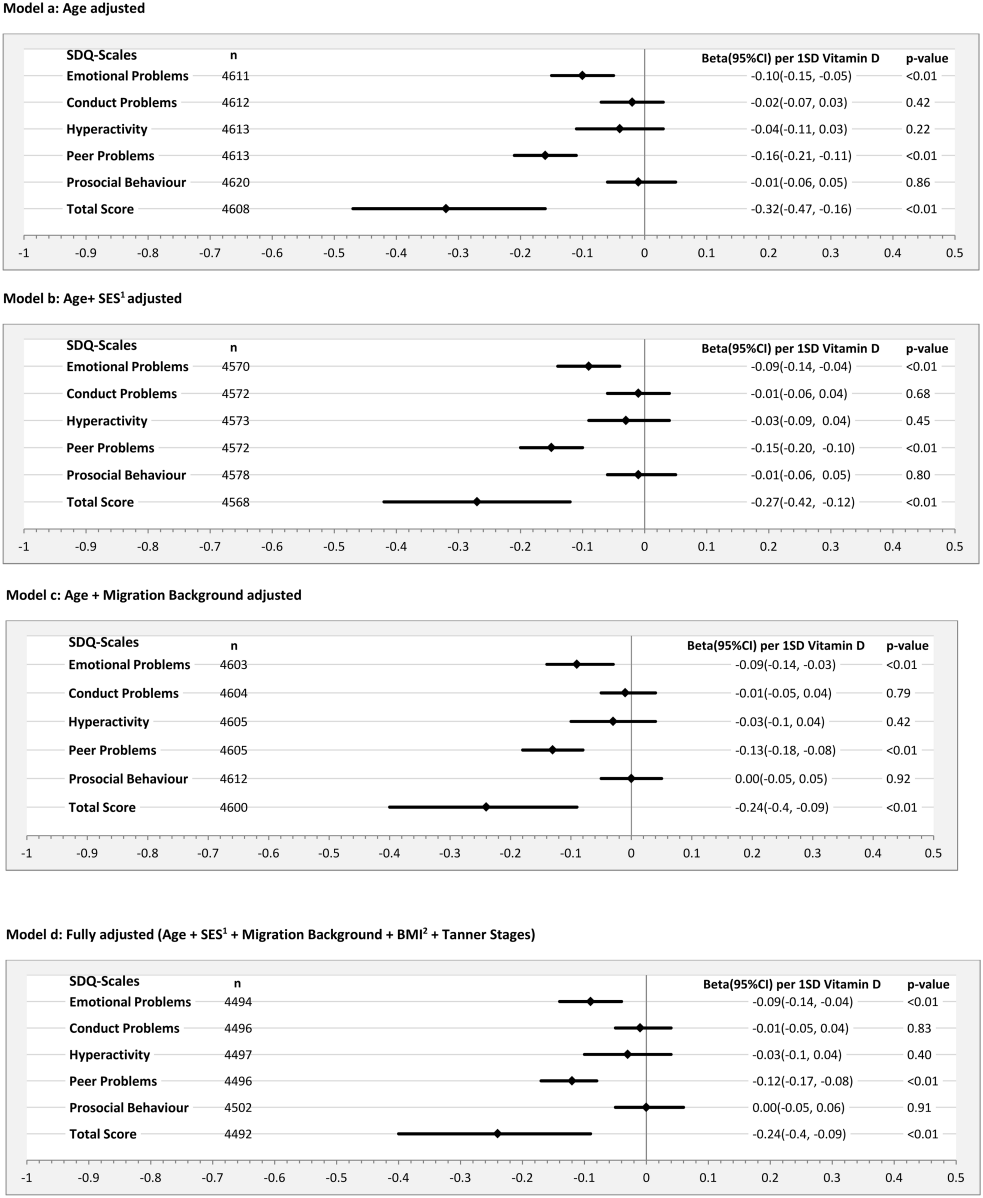
Beta estimates and corresponding 95% confidence intervals (95% CI) per standard deviation (SD = 25.0) increase of Vitamin D on Strengths and Difficulties Questionnaire (SDQ)-Subscales of the parent-ratings for boys aged 3–17 years using different adjusting sets in linear regression models. ^1^SES = socioeconomic status; ^2^BMI = Body Mass Index.

**Fig 2:**
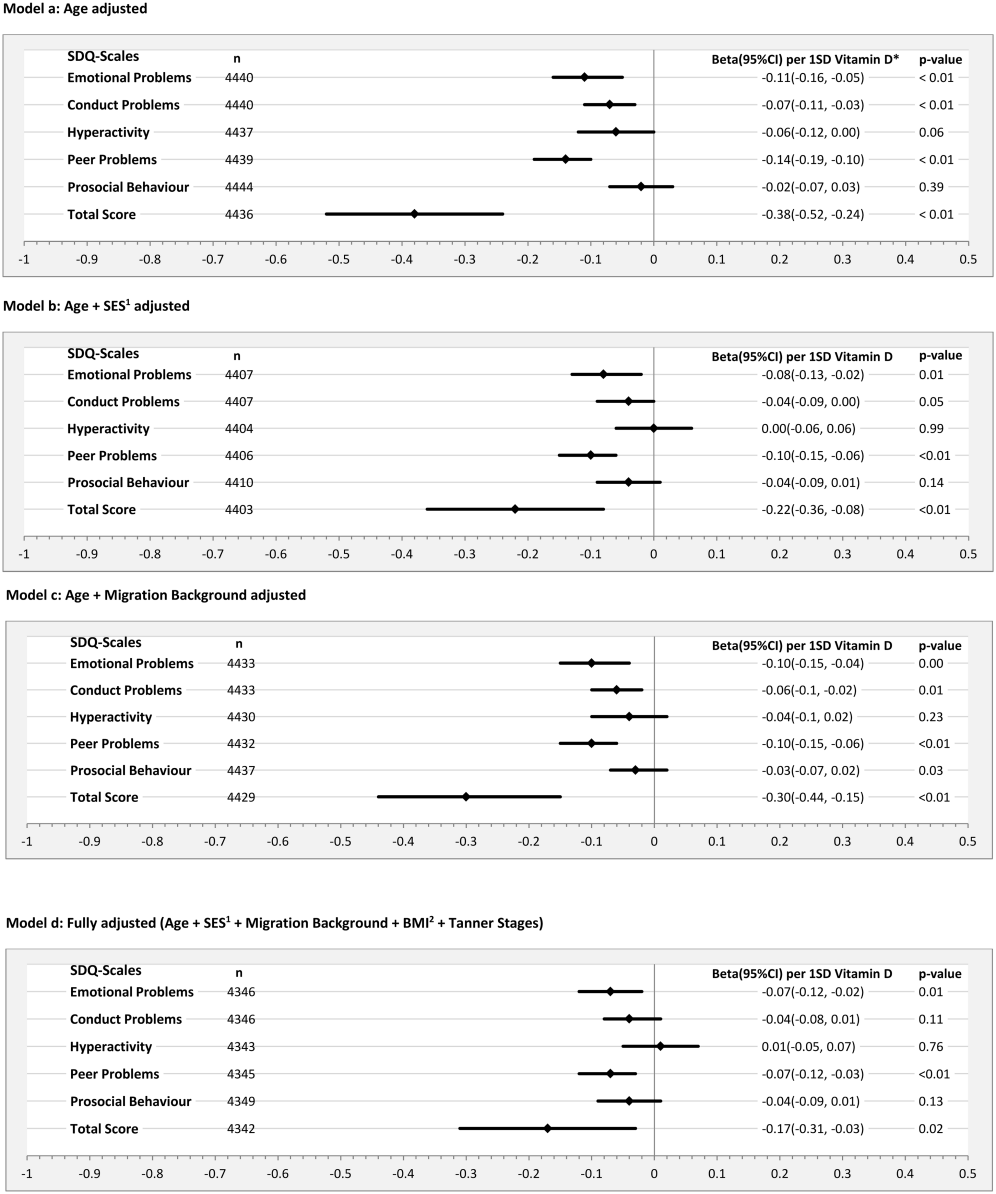
Beta estimates and corresponding 95% confidence intervals (95% CI) per standard deviation (SD = 25.5) increase of Vitamin D on Strength and Difficulties Questionnaire (SDQ)-Subscales of the parent-ratings for girls aged 3–17 years using different adjusting sets in linear regression models. ^1^SES = socioeconomic status; ^2^BMI = Body Mass Index.

Total sample (3–17 years): The highest effect size estimates were consistently found for the subscales emotional problems and peer relationship problems in all regression models. Effect size estimates were almost zero for the subscales conduct problems, prosocial problems and hyperactivity in the fully adjusted model [Figs 1 and 2, Model d]. An increase of one gender-specific standard deviation of the 25(OH)D serum concentration (boys: 25.0 nmol/l; girls: 25.5 nmol/l) was associated with a slight decrease in the SDQ total difficulties score while slightly stronger effects were observed in boys (-0.24 [Fig 1, Model d]) as compared to girls (-0.17 [Fig 2, Model d]).

Both the additional adjustments for SES (Model b) and migration status (Model c) led to reductions in effect size estimates (Figs 1 and 2). In boys adjustment for migration background (Model c) led to a stronger reduction in the effect size estimates for the total difficulties score than adjustment for SES (Model b). For girls the inverse was observed (Fig 2).

After adjusting for all covariates (Model d), effect size estimates remained basically unchanged. The decrease of the effect size estimate for the total difficulties score in the fully adjusted model as compared with the age adjusted model was more pronounced in girls than in boys (Figs 1 and 2).

Subsample analysis in the younger sample (3–11 years, for description see Supporting Information S1 Table): The effect size estimates were close to zero in the younger subsample; 95% confidence intervals included zero for all subscales and the total difficulties scores (S1 and S2 Figs). In boys and girls adjustment for migration background (Model c) and SES (Model b), respectively, reduced the effect size estimates the strongest. In the fully adjusted Model d the effect size estimates only marginally changed in boys and girls compared to Models b and c. After the additional adjustment for the dichotomous variable “frequency of playing outside” (Model e) in the fully adjusted model, the effect size estimates remained similar.

Subsample analysis in the older sample (12–17 years, for description see Supporting Information S2 Table): The effect size estimates in the older subsample (parent-ratings) were higher than in the younger subsample with regard to the fully adjusted model (95% confidence intervals overlap; for comparison see S3 and S5 Tables versus S1 and S2 Figs Model d). As in the total sample, the highest effect size estimates were found for emotional problems, peer relationship problems and the total difficulties score both in boys and girls. In boys, an increase of 24.6 nmol/L resulted in a decrease of 0.38 in the total difficulties score (S3 Table), in girls an increase of 26.7 nmol/L in a decrease of 0.19 respectively (S5 Table). In girls the final adjustment set including Tanner stages and BMI led to a further decrease of the effect size estimates.

The effect size estimates of emotional problems, peer relationship problems and total difficulties score were higher in parent-ratings than in self-ratings for boys (95% confidence intervals overlap; S3 and S4 Tables). With regard to the subscale peer relationship problems the 95% confidence intervals only slightly overlap. In girls, the effect size estimates of the respective subscales and the total difficulties score were higher in self-ratings; however, the overlap in the 95% confidence interval was large (S5 and S6 Tables, Model d). Compared to girls effect size estimates of peer relationship problems and total difficulties score were higher in boys with regard to parent-ratings (95% confidence intervals overlap; S3 and S5 Tables); in contrast self-ratings resulted in higher effect size estimates of emotional problems, peer relationship problems and total difficulties score in girls (S4 and S6 Tables; 95% confidence intervals overlap).

Within the fully adjusted model, SES revealed significant effects on the association between 25(OH)D level and SDQ total difficulties score in the older subsample with regard to self- and parent-ratings (S7 and S8 Tables).

## Discussion

### Associations between 25(OH)- vitamin D concentrations and the SDQ subscales

The current study revealed weak associations between 25(OH)D concentrations and the total difficulties scale of the SDQ in this first cross-sectional analysis based on a nation-wide population-based data set of a broad age range including 3–17.9 year old children and adolescents. Subscale analysis showed that the inverse association was mainly due to the subscales emotional problems and peer relationship problems even after adjustment for potential confounders/ mediators. The effect–albeit small—is in line with previously reported associations between vitamin D deficiency and mental health conditions [32, 37, 48, 49]. Depressive symptoms including emotional and peer relationship problems seem to be associated with vitamin D deficiency as previously shown for the 11–17 year old subsample [37].

Especially in adults evidence has accumulated for an inverse relationship of depressive symptoms and 25(OH)D levels [48, 49]. We found a rather stable association between peer relationship problems and 25(OH)D concentrations especially within the older study sample (12–17 years) compared to the younger subsample (3–11 years) after adjustment for potential confounders/ mediators. This scale includes being alone, being picked on/bullied, not being liked by other children and getting on better with adults than with other children perhaps indicating that affected children may have an inhibited temperament [50]. Because of the cross-sectional design, the question of direction of effects cannot be answered and reverse causation may be possible. Maybe children and adolescents with low 25(OH)D concentrations tend to withdraw from others, hence, making less friends and behaving cautiously or passively; this could render them more likely to be bullied. Vice versa, it is just as well possible that children/ adolescents, who are socially withdrawn, tend to be less active outside and therefore have lower 25(OH)D concentrations. If a child/adolescent withdraws from others, he/she might not be able to interact in a pro-social manner, because he/she does not have the chance to practice pro-social behavior.

Overall, a stronger association between 25(OH)D and SDQ-subscales was found in the older subsample. This finding might be explained by both the higher prevalence of depressive symptoms and of vitamin D deficiency in adolescence [42, 51]. At the age of 14 years, more than 50% of adolescents show a 25(OH)D level < 50nmol/l according to KiGGS. Recent data from a standardization of serum 25(OH)D data deriving from 18 nationally or regionally representative European studies according to international Vitamin D Standardization Program (VDSP) suggests that the risk of vitamin D deficiency is higher in adolescents than in children, adults, and the elderly (>61 years), although differences in latitude of sample population, ethnic mix, and season of blood sampling limit the validity of this finding [52]

The missing detection of significant longitudinal associations with respect to the SDQ in the cohort study of Tolppanen et al. (ALSPAC) might be due to the investigated age-span of 10 to 12 years and the lower prevalence of vitamin D deficiency [33]. Furthermore, the covariates included and the exclusion of children with behavioral problems at the age 7.6 or 9.6 might have influenced the results. In the ALSPAC study only a weak association between lower 25(OH)D_3_ (the major contributor to total 25(OH)D) and higher risk of prosocial problems was found, while the relationship between prosocial behavior (SDQ subscale) and prosocial problems was not delineated [33]. On the other hand it has to be noted that the same research group found the first prospective association between 25(OH)D concentrations and depressive symptoms [32] in childhood assessed by the Mood and Feelings Questionnaire. In addition, the missing associations in the ALSPAC study may reflect a type 2 error due to the small number of children with borderline or abnormal behavior [n = 86 (3.5%) for total difficulties, 171 (6.7%) for emotional symptoms, 255 (9.0%) for conduct problems 127 (4.9%) for hyperactivity, 197 (8.1%) for peer relationship problems and 101 (3.8%) for pro-social problems], particularly in light of the fact that a trend in the expected direction was apparent (odds ratios of approximately 0.9 in all three models; the 95% confidence intervals ranged from 0.75 to 1.02).

In another recent analysis from an adolescent subsample of the German KiGGS study population (14–17.9 years) the association between mental health and vitamin D deficiency was confirmed via a multifactorial logistic regression analysis, which revealed a five-fold higher rate of parent-reported mental service use due to children’s behavioral and emotional problems in severe vitamin D deficient children [53].

### The impact of the analyzed covariates

As SES and migration status reduced the effect size estimates obtained, parts of the age-adjusted association between mental health problems and 25(OH)D concentrations can be explained by confounding of with these variables. Migration status and SES are known to be associated, as children and adolescents with migration background in Germany are significantly more likely to have a lower SES than children and adolescents without a migration background [44]. Both low SES and migration background are correlated with mental health problems [39, 44] which could explain at least part of the influence of SES and migration status on the association between the SDQ subscales and 25(OH)D deficiency. Another possible explanation is that children with high SES might be more active outside and therefore have higher vitamin D concentrations. It is known that higher parental education level is correlated with higher physical activity levels of their offspring, at least in girls [54]. In addition, migration status often implies a stronger pigmented skin, which is correlated with a lowered 25(OH)D concentration [4].

In the young subsample we analyzed the effect of “frequency of playing outside” on the association between 25(OH)D and the SDQ subscales. The small effect size estimates remained similar. The lack of an effect is probably due to the young age in this subsample, so it was not possible to assess potential confounding by “frequency of playing outside”.

Our findings do suggest an impact of BMI and Tanner stages on the association between mental health problems and 25(OH)D in the older female subsample. The negative correlation between BMI and 25(OH)D concentration [4] and the positive correlation between Tanner stage and 25(OH)D concentration [41, 55] are well known as well as the association between BMI and mental health problems [56].

### Strengths and limitations

Strengths of our study are the large, nationwide population-based sample including both children and adolescents, the use of both self- and parent-ratings of mental health problems and the analysis of potential confounders that are hypothesized to play an important role in the association between mental health problems and 25(OH)D.

The main limitation is that “frequency of playing outside” was only available in a subsample and we were not able to include confounders such as UVB-protection, family history of schizophrenia and depression. The use of a cross-sectional design does not allow to draw conclusions related to the direction of the effects between low 25(OH)-vitamin D concentrations and mental health problems. Furthermore the population-based sample is naturally not “enriched” with patients with mental disorders and the SDQ is not a diagnostic interview and can thus not be used to analyze the association between 25(OH)-vitamin D concentrations and specific diagnostic entities like depression, autism, anxiety, schizophrenia.

Thus, clinical conclusions have to be drawn with caution. Overall, a clinical relevance cannot be delineated by the small effects detected. However, the associations were observed within regression models of different adjustment sets both in the total sample and the older subsample. However, due to the cross-sectional design and potential reverse causation direction of effects between 25(OH)D, SDQ-subscales and some of the covariates included in the analysis remains unclear. In addition, between-study differences in the sets of covariates adjusted for complicate a comparison of study results [57].

## Conclusions

Our study revealed associations between 25(OH)D concentrations and SDQ subscales such as emotional problems and peer relationship problems in children and adolescents after adjustment for potential confounders. In comparison to the previous study of Schäfer et al. [37] we demonstrate that the observed associations are stronger in adolescents and that the confounders migration background and SES reduced the effect size estimates most. Puberty and BMI seem to have an impact only in girls aged ≥12-<18. Longitudinal studies are required to confirm this finding. Especially interventional RCTs are a *conditio sine qua non* for delivering causal evidence; vitamin D supplementation of children with low 25(OH)D levels might represent a a simple, safe and cost-efficient extension of current therapeutic options for children and adolescents with emotional and behavioral problems.

## Supporting information

S1 Fig Beta estimates and corresponding 95% confidence intervals (95% CI) per standard deviation (SD = 25,2) increase of Vitamin D on Strength and Difficulties Questionnaire (SDQ)-subscales of the parent-ratings for boys aged 3–11 years using different adjusting sets including “frequency playing outside” in linear regression models. ^1^SES = socioeconomic status; ^2^BMI = Body Mass Index.
(DOCX)

S2 Fig Beta estimates and corresponding 95% confidence intervals (95% CI) per standard deviation (SD = 25.0) increase of Vitamin D on Strength and Difficulties Questionnaire (SDQ)-subscales of the parent-ratings for girls aged 3–11 years using different adjusting sets including “frequency playing outside” in linear regression models. ^1^SES = socioeconomic status; ^2^BMI = Body Mass Index.
(DOCX)

S1 Table Characteristics of sub-population aged 3–11 years. ^a^ n(%), ^b^Mean (Standard deviation).
(DOCX)

S2 Table Characteristics of study population aged 12–17 years. ^a^ n(%), ^b^Mean (Standard deviation).
(DOCX)

S3 Table Beta estimates and corresponding 95% confidence intervals (95% CI) per standard deviation (SD = 26.74) increase of Vitamin D on Strength and Difficulties Questionnaire (SDQ)-subscales of the parent-ratings for boys aged 12–17 years using different adjusting sets in linear regression models. *Values per 1 SD Vitamin D = 24.63, ^1^Vitamin D + Age, ^2^Model a + Socioeconomic Status (SES), ^3^Model a + Migration Background, ^4^ Fully adjusted (Age + SES + Migration Background + Body Mass Index + Tanner Stages).
(DOCX)

S4 Table Beta estimates and corresponding 95% confidence intervals (95% CI) per standard deviation (SD = 26.74) increase of Vitamin D on Strength and Difficulties Questionnaire (SDQ)-subscales of the self-ratings for boys aged 12–17 years using different adjusting sets in linear regression models. *Values per 1 SD Vitamin D = 24.63, ^1^Vitamin D + Age, ^2^Model a + Socioeconomic Status (SES), ^3^Model a + Migration Background, ^4^ Fully adjusted (Age + SES + Migration Background + Body Mass Index + Tanner Stages).
(DOCX)

S5 Table Beta estimates and corresponding 95% confidence intervals (95% CI) per standard deviation (SD = 26.74) increase of Vitamin D on Strength and Difficulties Questionnaire (SDQ)-subscales of the parent-ratings for girls aged 12–17 years using different adjusting sets in linear regression models. *Values per 1 SD Vitamin D = 26.74, ^1^Vitamin D + Age, ^2^Model a + Socioeconomic Status (SES), ^3^Model a + Migration Background, ^4^Fully adjusted (Age + SES + Migration Background + Body Mass Index + Tanner Stages).
(DOCX)

S6 Table Beta estimates and corresponding 95% confidence intervals (95% CI) per standard deviation (SD = 26.74) increase of Vitamin D on Strength and Difficulties Questionnaire (SDQ)-subscales of the self-ratings for girls aged 12–17 years using different adjusting sets in linear regression models. *Values per 1 SD Vitamin D = 26.74, ^1^Vitamin D + Age, ^2^Model a + Socioeconomic Status (SES), ^3^Model a + Migration Background, ^4^Fully adjusted (Age + SES + Migration Background + Body Mass Index + Tanner Stages).
(DOCX)

S7 Table Beta estimates and corresponding 95% confidence intervals (95% CI) for the association of Vitamin D on the SDQ total difficulties score for boys aged 12–17 years with regard to parent- and self-ratings using the full adjustment set (age + SES + migration background + body mass index + tanner stages). *Socioeconomic Status = SES.
(DOCX)

S8 Table Beta estimates and corresponding 95% confidence intervals (95% CI) for the association of Vitamin D on the SDQ total difficulties score for girls aged 12–17 years with regard to parent- and self-ratings using the full adjustment set (age + SES + migration background + body mass index + tanner stages). *Socioeconomic Status = SES.
(DOCX)
