# Supplementary material for: Low 25(OH)-vitamin D concentrations are associated with emotional and behavioral problems in German children and adolescents
Source: PLoS One. 2017 Aug 23;12(8):e0183091. doi: 10.1371/journal.pone.0183091 (PMC5568331; doi:10.1371/journal.pone.0183091)
Supplement: S1 Fig — 1SES = socioeconomic status; 2BMI = Body Mass Index. (DOCX) [file pone.0183091.s001.docx]

**S1 Fig. Beta estimates and corresponding 95% confidence intervals (95% CI) per standard deviation (SD=25,2) increase of Vitamin D on Strengths and Difficulties Questionnaire (SDQ)-Subscales of the parent-ratings for boys aged 3-11 years using different adjusting sets including “Frequency Playing Outside” in linear regression models.**

**Model d: Fully adjusted (Age + SES^1^ + Migration Background + BMI^2^ + Tanner Stages)**

**Model e: Fully adjusted (Age + SES^1^ + Migration Background + BMI^2^ + Tanner Stages) + Frequency Playing Outside**
