# Supplementary material for: Low 25(OH)-vitamin D concentrations are associated with emotional and behavioral problems in German children and adolescents
Source: PLoS One. 2017 Aug 23;12(8):e0183091. doi: 10.1371/journal.pone.0183091 (PMC5568331; doi:10.1371/journal.pone.0183091)
Supplement: S1 Table — a n(%), bMean (Standard deviation). (DOCX) [file pone.0183091.s003.docx]

**S1 Table. Characteristics of sub-population aged 3-11 years.**

|  | | | **Boys** | | **Girls** | |
| --- | --- | --- | --- | --- | --- | --- |
| **N** | | | 2358 | | 2274 | |
| **25(OH)Vitamin D [nmol/l] ^b^** | | | 49.7 ± 25.2 | | 48.4 ± 25.0 | |
| **Strengths and Difficulties Questionnaire, Parent-Ratings^b^** | | |  | |  | |
| Emotional Problems | | | 1.7 ± 1.8 | | 1.8 ±1.7 | |
| Conduct Problems | | | 2.1 ± 1.5 | | 1.8 ±1.4 | |
| Hyperactivity | | | 3.7 ± 2.4 | | 3.0 ±2.2 | |
| Peer Relationship Problems | | | 1.4 ± 1.6 | | 1.2 ±1.5 | |
| Prosocial Behaviour | | | 7.6 ± 1.7 | | 8.1 ±1.6 | |
| Total Difficulties Score | | | 8.9 ± 5.2 | | 7.7 ±4.8 | |
| **Socioeconomic Status^a^** | | |  | |  | |
| Low | | | 258 (28.0%) | | 616 (27.1%) | |
| Middle | | | 1082 (46.0%) | | 1051 (46.3%) | |
| High | | | 613 (26.1%) | | 603 (26.6%) | |
| **Migrant Background^a^** | | | 296 (12.6%) | | 270 (11.9%) | |
| **Body Mass Index ^a^** | | |  | |  | |
| Severely Underweight (<P3) | | | 38 (1.6%) | | 37 (1.6%) | |
| Underweight (P3- <P10) | | | 120 (5.1%) | | 116 (5.1%) | |
| Normal (Healthy Weight) | | | 1896 (80.6%) | | 1836 (81.0%) | |
| Overweight (>P90 – P97) | | | 177 (7.5%) | | 179 (7.9%) | |
| Obese (>P97) | | | 120 (5.1%) | | 99 (4.4%) | |
| **Development of Pubic Hair (Tanner Stages)^a^** | 1 | | 2231 (94.6%) | | 2142 (94.6%) | |
|  | 2 | | 102 (4.3%) | | 84 (3.7%) | |
|  | 3 | | 13 (0.6%) | | 17 (0.8%) | |
|  | 4 | | 4 (0.2%) | | 22 (1.0%) | |
|  | 5 | | 0 | | 0 | |
|  | 6 | | 0 | | 0 | |
| **Frequency**  **Playing Outside ^a^** | Almost daily | | 1846 (78.3%) | | 1697 (74.6%) | |
|  | 3-5 times / Week | | 382 (16.2%) | | 420 (18.5%) | |
|  | 1-2 times / Week | | 98 (4.2%) | | 125 (5.5%) | |
|  | Seldom / Never | | 32 (1.4%) | | 32 (1.4%) | |
| ^a^ n(%) | |  |  |  | |  |
| ^b^ Mean (Standard deviation) | |  |  |  | |  |
|  | | | | | | |
