# Supplementary material for: Low 25(OH)-vitamin D concentrations are associated with emotional and behavioral problems in German children and adolescents
Source: PLoS One. 2017 Aug 23;12(8):e0183091. doi: 10.1371/journal.pone.0183091 (PMC5568331; doi:10.1371/journal.pone.0183091)
Supplement: S2 Table — a n(%), bMean (Standard deviation). (DOCX) [file pone.0183091.s004.docx]

**S2 Table. Characteristics of study population aged 12-17 years.**

|  | | **Boys** | **Girls** |
| --- | --- | --- | --- |
| **N** | | 1884 (50.9%) | 1819 (49.1%) |
| **25(OH)Vitamin D [nmol/l] ^b^** | | 44.2 ± 24.6 | 45.7 ± 26.7 |
| **Strength and Difficulties Questionnaire, Parental-Ratings^b^** | |  |  |
| Emotional Problems | | 1.5 ± 1.8 | 1.9 ± 1.8 |
| Conduct Problems | | 2.0 ± 1.6 | 1.8 ± 1.5 |
| Hyperactivity | | 3.2 ± 2.2 | 2.3 ± 1.9 |
| Peer Relationship Problems | | 1.6 ± 1.7 | 1.4 ± 1.5 |
| Prosocial Behaviour | | 7.5 ± 1.8 | 8.0 ± 1.7 |
| Total Difficulties Score | | 8.3 ± 5.3 | 7.3 ± 4.8 |
| **Strengths and Difficulties Questionnaire, Self-Ratings ^b^** | |  |  |
| Emotional Problems | | 1.9 ± 1.6 | 3.0 ± 2.1 |
| Conduct Problems | | 2.0 ± 1.4 | 1.9 ± 1.3 |
| Hyperactivity | | 3.6 ± 2.1 | 3.6 ± 2.0 |
| Peer Relationship Problems | | 1.9 ± 1.5 | 2.0 ± 1.5 |
| Prosocial Behaviour | | 7.2 ± 1.8 | 8.2 ± 1.5 |
| Total Difficulties Score | | 9.4 ± 4.3 | 10.6 ± 4.6 |
| **Socioeconomic Status^a^** | |  |  |
| Low | | 507 (27.2%) | 485 (26.9%) |
| Middle | | 909 (48.8%) | 863 (47.9%) |
| High | | 448 (24.0%) | 455 (25.2%) |
| **Migrant Background^a^** | | 260 (13.8%) | 249 (13.7%) |
| **Body Mass Index ^a^** | |  |  |
| Severely Underweight (<P3) | | 48 (2.6%) | 33 (1.8%) |
| Underweight (P3- <P10) | | 106 (5.7%) | 91 (5.0%) |
| Normal (Healthy Weight) | | 1383 (73.8% | 1380 (76.3%) |
| Overweight (>P90 – P97) | | 196 (10.5%) | 160 (8.8%) |
| Obese (>P97) | | 140 (7.5%) | 146 (8.1%) |
| **Growth of Pubic Hair (Tanner Stages)^a^** | 1 | 38 (2.1%) | 35 (2.0%) |
|  | 2 | 221 (11.9%) | 57 (3.2%) |
|  | 3 | 215 (11.6%) | 113 (6.3%) |
|  | 4 | 325 (17.6%) | 477 (26.6%) |
|  | 5 | 716 (38.7%) | 858 (47.9%) |
|  | 6 | 336 (18.1%) | 253 (14.0%) |

^a^ n (%)

^b^ Mean (Standard deviation)
