# Supplementary material for: Low 25(OH)-vitamin D concentrations are associated with emotional and behavioral problems in German children and adolescents
Source: PLoS One. 2017 Aug 23;12(8):e0183091. doi: 10.1371/journal.pone.0183091 (PMC5568331; doi:10.1371/journal.pone.0183091)
Supplement: S3 Table — *Values per 1 SD Vitamin D = 24.63, 1Vitamin D + Age, 2Model a + Socioeconomic Status (SES), 3Model a + Migration Background, 4 Fully adjusted (Age + SES + Migration Background + Body Mass Index + Tanner Stages). (DOCX) [file pone.0183091.s005.docx]

**S3 Table. Beta estimates and corresponding 95% confidence intervals (95% CI) per standard deviation (SD= 26.74) increase of Vitamin D on Strengths and Difficulties Questionnaire (SDQ)-Subscales of the parent-ratings for boys aged 12-17 years using different adjusting sets in linear regression models.**

| **SDQ =** | **Model a^1^** | | | | | **Model b^2^** | | | | | **Model c^3^** | | | | | **Model d^4^** | | | | |
| --- | --- | --- | --- | --- | --- | --- | --- | --- | --- | --- | --- | --- | --- | --- | --- | --- | --- | --- | --- | --- |
|  | n | Beta* | 95% CI* | | p-value | n | Beta* | 95% CI* | | p-value | n | Beta* | 95% CI* | | p-value | n | Beta* | 95% CI* | | p-value |
|  |  |  | Lower | Upper |  |  |  | Lower | Upper |  |  |  | Lower | Upper |  |  |  | Lower | Upper |  |
| Emotional Problems | 1877 | -0.12 | -0.20 | -0.04 | 0.00 | 1859 | -0.11 | -0.20 | -0.02 | 0.01 | 1877 | -0.11 | -0.19 | -0.03 | 0.01 | 1816 | -0.11 | -0.19 | -0.03 | 0.01 |
| Conduct Problems | 1877 | -0.02 | -0.09 | 0.06 | 0.68 | 1860 | -0.01 | -0.08 | 0.07 | 0.82 | 1877 | -0.01 | -0.08 | 0.06 | 0.78 | 1817 | -0.01 | -0.09 | 0.06 | 0.76 |
| Hyperactivity | 1877 | -0.07 | -0.17 | 0.03 | 0.18 | 1860 | -0.05 | -0.15 | 0.05 | 0.29 | 1877 | -0.06 | -0.17 | 0.04 | 0.21 | 1817 | -0.06 | -0.16 | 0.04 | 0.25 |
| Peer Relationship Problems | 1878 | -0.23 | -0.31 | -0.16 | 0.00 | 1860 | -0.22 | -0.30 | -0.15 | 0.00 | 1878 | -0.21 | -0.29 | -0.14 | 0.00 | 1817 | -0.20 | -0.28 | -0.12 | 0.00 |
| Prosocial Behaviour | 1883 | 0.00 | -0.08 | 0.08 | 1.00 | 1864 | 0.00 | -0.09 | 0.08 | 0.91 | 1883 | 0.01 | -0.07 | 0.09 | 0.80 | 1821 | 0.01 | -0.08 | 0.09 | 0.85 |
| Total Difficulties Score | 1875 | -0.43 | -0.67 | -00.19 | 0.00 | 1858 | -0.39 | -0.62 | -0.15 | 0.00 | 1875 | -0.39 | -0.63 | -0.15 | 0.00 | 1815 | -0.38 | -0.62 | -0.14 | 0.00 |

* Values per 1 SD Vitamin D= 24.63

^1^ Vitamin D + Age

^2^ Model a + Socioeconomic Status (SES)

^3^ Model a + Migration Background

^4^ Fully adjusted (Age + SES + Migration Background + Body Mass Index + Tanner Stages)
