# Supplementary material for: Low 25(OH)-vitamin D concentrations are associated with emotional and behavioral problems in German children and adolescents
Source: PLoS One. 2017 Aug 23;12(8):e0183091. doi: 10.1371/journal.pone.0183091 (PMC5568331; doi:10.1371/journal.pone.0183091)
Supplement: S4 Table — *Values per 1 SD Vitamin D = 24.63, 1Vitamin D + Age, 2Model a + Socioeconomic Status (SES), 3Model a + Migration Background, 4 Fully adjusted (Age + SES + Migration Background + Body Mass Index + Tanner Stages). (DOCX) [file pone.0183091.s006.docx]

**S4 Table. Beta estimates and corresponding 95% confidence intervals (95% CI) per standard deviation (SD= 26.74) increase of Vitamin D on Strengths and Difficulties Questionnaire (SDQ)-Subscales of the self-ratings for boys aged 12-17 years using different adjusting sets in linear regression models.**

| **SDQ =** | **Model a^1^** | | | | | **Model b^2^** | | | | | **Model c^3^** | | | | | **Model d^4^** | | | | |
| --- | --- | --- | --- | --- | --- | --- | --- | --- | --- | --- | --- | --- | --- | --- | --- | --- | --- | --- | --- | --- |
|  | n | Beta* | 95% CI* | | p-value | n | Beta* | 95% CI* | | p-value | n | Beta* | 95% CI* | | p-value | n | Beta* | 95% CI* | | p-value |
|  |  |  | Lower | Upper |  |  |  | Lower | Upper |  |  |  | Lower | Upper |  |  |  | Lower | Upper |  |
| Emotional Problems | 1863 | -0.06 | -0.13 | 0.01 | 0.11 | 1846 | -0.06 | -0.13 | 0.02 | 0.12 | 1863 | -0.06 | -0.13 | 0.02 | 0.12 | 1804 | -0.05 | -0.12 | 0.02 | 0.19 |
| Conduct Problems | 1863 | -0.02 | -0.09 | 0.04 | 0.48 | 1846 | -0.02 | -0.09 | 0.04 | 0.51 | 1863 | -0.02 | -0.09 | 0.05 | 0.55 | 1804 | -0.02 | -0.09 | 0.04 | 0.48 |
| Hyperactivity | 1863 | 0.04 | -0.05 | 0.13 | 0.41 | 1846 | 0.05 | -0.05 | 0.14 | 0.31 | 1863 | 0.03 | -0.06 | 0.13 | 0.48 | 1804 | 0.04 | -0.05 | 0.14 | 0.39 |
| Peer Relationship Problems | 1863 | -0.07 | -0.13 | 0.00 | 0.06 | 1846 | -0.06 | -0.13 | 0.01 | 0.07 | 1863 | -0.06 | -0.13 | 0.01 | 0.08 | 1804 | -0.05 | -0.12 | 0.02 | 0.13 |
| Prosocial Behaviour | 1862 | -0.05 | -0.13 | 0.03 | 0.19 | 1845 | -0.05 | -0.13 | 0.00 | 0.22 | 1862 | -0.04 | -0.12 | 0.04 | 0.30 | 1803 | -0.03 | -0.11 | 0.06 | 0.54 |
| Total Difficulties Score | 1863 | -0.11 | -0.31 | 0.09 | 0.28 | 1846 | -0.09 | -0.29 | 0.10 | 0.35 | 1863 | -0.11 | -0.30 | 0.09 | 0.29 | 1804 | -0.08 | -0.28 | 0.11 | 0.40 |

* Values per 1 SD Vitamin D= 24.63

^1^ Vitamin D + Age

^2^ Model a + Socioeconomic Status (SES)

^3^ Model a + Migration Background

^4^ Fully adjusted (Age + SES + Migration Background + Body Mass Index + Tanner Stages)
