# Supplementary material for: Low 25(OH)-vitamin D concentrations are associated with emotional and behavioral problems in German children and adolescents
Source: PLoS One. 2017 Aug 23;12(8):e0183091. doi: 10.1371/journal.pone.0183091 (PMC5568331; doi:10.1371/journal.pone.0183091)
Supplement: S7 Table — *Socioeconomic Status = SES. (DOCX) [file pone.0183091.s009.docx]

**S7 Table: Beta estimates and corresponding 95% confidence intervals (95% CI) for the association of Vitamin D on the SDQ total difficulties score for boys aged 12-17 years with regard to parent- and self-ratings using the full adjustment set (age + socioeconomic status + migration background + body mass index + Tanner stages)**

|  |  | SDQ total difficulties score: boys/parent-rating | | | | SDQ total difficulties score: boys/self-rating | | | |
| --- | --- | --- | --- | --- | --- | --- | --- | --- | --- |
|  |  | Beta | 95% CI | | p-value | Beta | 95% CI | | p-value |
|  |  |  | Lower | Upper |  |  | Lower | Upper |  |
| 25(OH)D [nmol/L] (per SD) |  | -0.37 | -0.62 | -0.15 | <0.01 | -0.10 | -0.30 | 0.12 | 0.39 |
| Age (years) | 12-13 | Ref.- |  |  |  | Ref.- |  |  |  |
|  | 14-15 | -0.88 | -1.53 | -0.23 | <0.01 | -0.05 | -0.59 | 0.49 | 0.86 |
|  | 16-17 | -1.14 | -1.86 | -0.43 | <0.01 | 0.40 | -0.20 | 1.00 | 0.19 |
| SES* (per score unit Winkler-Index) |  | -0.22 | -0.28 | -0.17 | <0.01 | -0.16 | -0.21 | -0.11 | <0.01 |
| Migration Background |  | -0.24 | -0.98 | 0.50 | 0.53 | 0.49 | -0.12 | 1.11 | 0.12 |
| Body mass index (BMI) | Normal Weight | Ref.- |  |  |  | Ref.- |  |  |  |
|  | Obese (>P97) | 0.51 | -0.43 | 1.45 | 0.28 | 1.10 | 0.32 | 1.88 | 0.01 |
|  | Overweight (>P90) | 0.22 | -0.57 | 1.01 | 0.58 | 0.53 | -0.13 | 1.18 | 0.12 |
|  | Severely Underweight (<P3) | 0.21 | -1.30 | 1.71 | 0.79 | -1.05 | -2.30 | 0.20 | 0.10 |
|  | Underweight (P3-<P10) | -0.41 | -1.43 | 0.62 | 0.43 | -0.08 | -0.93 | 0.77 | 0.85 |
| Development of Pubic Hair (Tanner Stages) | Prepubescent (Tanner Stages 1-3) | Ref.- |  |  |  | Ref.- |  |  |  |
|  | Pubescent (Tanner Stages 4-6) | -0.42 | -1.10 | 0.26 | 0.22 | -0.74 | -1.30 | -0.17 | 0.01 |
