# Supplementary material for: Low 25(OH)-vitamin D concentrations are associated with emotional and behavioral problems in German children and adolescents
Source: PLoS One. 2017 Aug 23;12(8):e0183091. doi: 10.1371/journal.pone.0183091 (PMC5568331; doi:10.1371/journal.pone.0183091)
Supplement: S8 Table — *Socioeconomic Status = SES. (DOCX) [file pone.0183091.s010.docx]

**S8 Table: Beta estimates and corresponding 95% confidence intervals (95% CI) for the association of Vitamin D on the SDQ total difficulties score for girls aged 12-17 years with regard to parent- and self-ratings using the full adjustment set (age + socioeconomic status + migration background + body mass index + Tanner stages)**

|  |  | SDQ total difficulties score: girls/parent-rating | | | | SDQ total difficulties score: girls/self-rating | | | |
| --- | --- | --- | --- | --- | --- | --- | --- | --- | --- |
|  |  | Beta | 95% CI | | p-value | Beta | 95% CI | | p-value |
|  |  |  | Lower | Upper |  |  | Lower | Upper |  |
| 25(OH)D [nmol/L] (per SD) |  | -0.19 | -0.40 | 0.03 | 0.11 | -0.24 | -0.45 | -0.03 | 0.04 |
| Age | 12-13 | Ref.- |  |  |  | Ref.- |  |  |  |
|  | 14-15 | -0.41 | -0.96 | 0.14 | 0.14 | 0.57 | 0.03 | 1.10 | 0.04 |
|  | 16-17 | -1.2 | -1.70 | -0.54 | <0.01 | 0.21 | -0.35 | 0.78 | 0.46 |
| SES* (per score unit Winkler-Index) |  | -0.22 | -0.28 | -0.17 | <0.01 | -0.10 | -0.15 | -0.05 | <0.01 |
| Migration Background |  | -1.24 | -1.90 | -0.59 | <0.01 | -0.76 | -1.39 | -0.12 | 0.02 |
| Body mass index (BMI) | Normal Weight | Ref.- |  |  |  | Ref.- |  |  |  |
|  | Obese (>P97) | 1.35 | 0.54 | 2.16 | <0.01 | 1.43 | 0.64 | 2.22 | <0.01 |
|  | Overweight (>P90) | 1.04 | 0.26 | 1.81 | 0.01 | 0.92 | 0.17 | 1.67 | 0.02 |
|  | Severely Underweight (<P3) | 1.84 | 0.20 | 3.48 | 0.03 | 0.33 | -1.26 | 1.93 | 0.68 |
|  | Underweight (P3-<P10) | 0.87 | -0.14 | 1.88 | 0.09 | 0.35 | -0.64 | 1.34 | 0.49 |
| Development of Pubic Hair (Tanner Stages) | Prepubescent (Tanner Stages 1-3) | Ref.- |  |  |  | Ref.- |  |  |  |
|  | Pubescent (Tanner Stages 4-6) | 0.54 | -0.20 | 1.28 | 0.15 | 0.49 | -0.23 | 1.21 | 0.18 |
